# A Direct Comparison of rAAV5 Variants Derived from the Baculovirus Expression System Using LC-MS Workflows Demonstrates Key Differences in Overall Production Yield, Product Quality and Vector Efficiency

**DOI:** 10.3390/ijms25052785

**Published:** 2024-02-28

**Authors:** Felipe Guapo, Nicholas Donohue, Lisa Strasser, Stefano Boi, Florian Füssl, Alana Rainbow-Fletcher, Paul Getty, Ian Anderson, Niall Barron, Jonathan Bones

**Affiliations:** 1 National Institute for Bioprocessing Research and Training, Foster Avenue, Mount Merrion, Blackrock, A94 X099 Dublin, Ireland; 2 Pharmaron, 12 Estuary Banks, Speke, Liverpool L24 8RB, UK; 3 School of Chemical and Bioprocess Engineering, University College Dublin, Belfield, Dublin 4, D04 V1W8 Dublin, Ireland

**Keywords:** AAV production, product quality analysis, LC-MS, critical quality attributes, gene therapy, process optimization, Sf9 cells

## Abstract

Gene therapy holds great promise for the treatment of severe diseases, and adeno-associated virus (AAV) vectors have emerged as valuable tools in this field. However, challenges such as immunogenicity and high production costs complicate the commercial viability of AAV-based therapies. To overcome these barriers, improvements in production yield, driven through the availability of robust and sensitive characterization techniques that allow for the monitoring of critical quality attributes to deepen product and process understanding are crucial. Among the main attributes affecting viral production and performance, the ratio between empty and full capsids along with capsid protein stoichiometry are emerging as potential parameters affecting product quality and safety. This study focused on the production of AAV vectors using the baculovirus expression vector system (BEVS) in Sf9 cells and the complete characterization of AAV5 variants using novel liquid chromatography and mass spectrometry techniques (LC-MS) that, up to this point, had only been applied to reference commercially produced virions. When comparing virions produced using ATG, CTG or ACG start codons of the *cap* gene, we determined that although ACG was the most productive in terms of virus yield, it was also the least effective in transducing mammalian cells. This correlated with a low VP1/VP2 ratio and a higher percentage of empty capsids. Overall, this study provides insights into the impact of translational start codon modifications during rAAV5 production using the BEVS, the associated relationship with capsid packaging, capsid protein stoichiometry and potency. The developed characterization workflow using LC-MS offers a comprehensive and transferable analysis of AAV-based gene therapies, with the potential to aid in process optimization and facilitate the large-scale commercial manufacturing of these promising treatments.

## 1. Introduction

Gene therapy is a rapidly evolving field that offers exciting potential for treating severe diseases [1,2]. Using selected vectors, a therapeutic gene can be delivered to patients with a single dose, triggering lifelong beneficial effects in many cases. Recombinant adeno-associated virus (rAAV) is used as a viral vector in gene therapy in an ever-increasing number of clinical trials. The first approved gene therapy medicine in Europe was the rAAV-based alipogene tiparvovec (Glybera^®^, uniQure, Amsterdam, The Netherlands), which received approval in 2012 but was later discontinued [3,4]. rAAV-based gene therapies have gained considerable interest in recent years [5,6,7,8] and many more commercial products are reaching the market. Concerns about immunogenicity and high costs associated with the production of such vectors complicate the commercial viability of rAAV-based gene therapy, representing critical barriers that need to be overcome for the broader success of this therapeutic format [4]. Thus, there is an urgent need to significantly improve the production yield and sensitivity of the main characterization techniques currently employed [9].

The baculovirus expression vector system (BEVS) has been successfully used for the expression of heterologous proteins and has proven to be effective in the industrial-scale production of rAAV [5]. Its use as a helper and delivery system for rAAV production in insect cells was first reported by Urabe et al. [6]. Further advancements using chimeric versions of AAVs in Sf9 insect cells allowed for higher production titers previously only seen in mammalian HEK293T cells, with the resulting vectors showing similar potency to wild-type virus (WT) [7]. The baculovirus system can be employed in different configurations: the ThreeBac approach uses one baculovirus containing the AAV gene *rep* (required for replication), another containing *cap* (capsid) and a final virus to deliver the therapeutic transgene [6]. In the TwoBac system, *rep* and *cap* are combined in one virus and the transgene is provided on a separate virus [10]. Finally, the OneBac system combines *rep*, *cap* and the transgene in a single virus [11]. The effective production of transduction-competent rAAV capsids in insect cells is directly dependent not only on appropriate expression levels of baculoviral proteins but also on the optimal expression of AAV Cap and Rep proteins [8]. Capsid proteins include viral proteins VP1, VP2 and VP3, which are generated via alternative splicing and differential codon usage of a single capsid ORF in the AAV genome [6,7,10]. This generates a theoretical protein expression ratio of 1:1:10, which is required for optimal capsid assembly. In the case of wild-type AAV2, the start codon for VP1 is ATG. However, this results in non-functional AAVs when used in the Sf9/baculovirus system as it fails to produce AAV vectors with the correct VP stoichiometry due to differences in the ribosomal machinery in insect compared to mammalian cells [8].

To address this issue, various approaches have been explored. For example, changing the start codon to ACG and the inclusion of a recognition sequence containing nine nucleotides upstream of the translation initiation start site improved the expression of functional AAV vectors [10]. Additionally, Kondratov et al. identified a ‘consensus’ Kozak sequence by comparing different AAV serotypes. This sequence contains TGTTTT followed by the ATG start codon [12] and successfully produces functional viral vectors. Bosma et al. tested yet another approach by changing the VP1 start codon to CTG and adding a GCT triplet after the start codon. This provided a more optimal downstream context, as a G base right after the start codon is known to improve translation efficiency [8].

The commercialization of AAV-based gene therapy products requires a better understanding and improvement of the production process as well as developing state-of-the-art characterization techniques with the robustness and sensitivity to correctly report critical product quality attributes such as titer, product safety and potency [9]. AAV viral effectiveness or potency as well as transduction efficiency appear to be directly correlated with VP stoichiometry [13,14,15].

**Figure 1:**
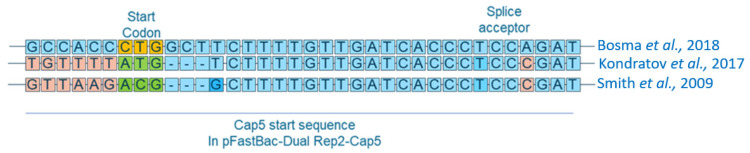
rAAV5 constructs previously developed by Smith et al. [10], Kondratov et al. [12] and Bosma et al. [8]. Smith’s construct incorporates an ACG start codon and a nine-nucleotide-long initiation sequence upstream of the translation initiation site. Kondratov’s contains an attenuated Kozak sequence TGTTTT followed by the ATG start codon and Bosma’s construct contains a CTG start codon followed by a GCT triplet.

In this report, we describe a complete characterization workflow for the in-depth analysis of rAAV variants using a series of novel liquid chromatography (LC) and mass spectrometry (MS) techniques. We comprehensively characterized in-house-produced variants of rAAV serotype 5 derived from Sf9 cells using the TwoBac system. Furthermore, we compared the effects of the *cap* start codon changes mentioned above by generating expression vectors based on the best-performing variants described by Smith et al. [10], Kondratov et al. [12] and Bosma et al. [8]. The sequences used to generate each rAAV are displayed in Figure 1. The chosen start codons (ACG, ATG, CTG) were found to perform well for AAV production in the original studies and our aim was to perform a direct comparison of the three variants under the same process conditions.

Product quality attribute profiles generated from our analyses were subsequently correlated to results from functional studies, which emphasized the benefits of using highly sensitive LC and MS techniques for the characterization of viral products over other traditional methods.

## 2. Results and Discussion

To monitor the different characteristics of rAAV5 capsid variants, sequence changes around the translational start of VP1 (*cap5*) were generated, as described in detail in Section 3. Sf9 cells were infected with baculovirus (BV) and cultures were harvested 72 h later. As expected, rAAV5 capsids were present in all samples, i.e., the supernatant (SUP) and cell pellet (CP) fractions. These were purified and assessed for total yield, transduction efficiency, VP ratio and ratio of full (containing a genome) to empty capsids, which represent important product quality attributes (PQAs) of AAV-based gene therapies. The production of rAAV5 using the baculovirus/Sf9 system is likely to lead to the production of further baculoviruses as well. To measure potential contamination, a negative control was included: Sf9 cells were transduced with just the transgene-containing baculovirus. This control could generate the transgene-containing baculovirus, but not rAAV5 due to the absence of *rep* and *cap*. The qPCR results from this sample indicated that contamination with baculovirus or other contaminating DNA was <1% of the yield of the experimental samples. Furthermore, a transduction assay in CHO-K1 cells using the negative control yielded < 1% transgene-positive cells.

### 2.1. Yield and Transduction Efficiency

The level of gene of interest (GOI)-containing encapsidated nucleic acid was measured by qPCR, while AAV viral particles were measured by ELISA. Transduction efficiency was determined by transducing CHO-K1 cells and then measuring %GFP-positive cells after 72 h using flow cytometry. CHO-K1 cells were chosen because the AAV5 serotype has a very limited tropism and CHO cells have been reported to be more easily transduced than many human cell lines [16].

**Figure 2:**
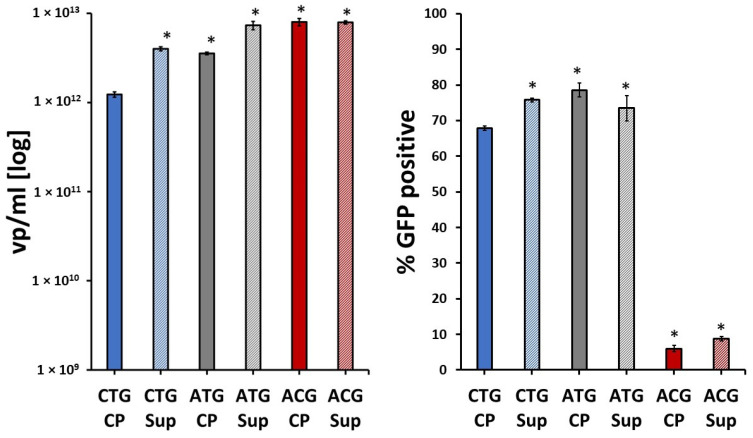
Comparison of viral particle titers and transduction efficiency of rAAV5 *cap* start codon variants. Samples were extracted from either cell pellet (CP) or supernatant (Sup) fractions. (**Left**) Total titer/yield measured by capsid ELISA post-purification for recombinant and wild-type AAV5 using the Progen AAV5 kit (Progen, Heidelberg, Germany). qPCR measurements are available in the Supplementary Materials (Figure S1). Error bars represent pooled samples derived from biological duplicates, with four technical replicates. (**Right**) In vitro transduction efficiency measurements of wild-type (ATG) and variant capsids in CHO-K1 cells. Two separate transductions were performed for each sample, with two technical repeats per transduction. One way ANOVA was run for each dataset individually using GraphPad Prism 5, followed by a post hoc Tukey’s multiple comparison test. Statistically significant values are identified as (*) *p* < 0.05 in comparison with CTG CP. For all individual comparisons, refer to statistical analysis results in the Supplementary Materials.

Upon analysis, rAAV produced with the ACG *cap* start codon showed the highest viral capsid titer, surpassing production from the ATG and CTG start codon variants. This was particularly evident in the cell pellet-derived samples. Interestingly, notwithstanding this high yield, the transduction efficiency of the ACG variant was by far the lowest of all three AAV variants (Figure 2).

The sequence variations surrounding the translational start of VP1 were generated to vary the stoichiometry of the three VPs expressed in the insect cells. The data demonstrate that these variants do indeed have an impact on overall productivity in Sf9 cells, but also on the potency of the resulting virus. Although these constructs have not been directly compared in terms of productivity and transduction efficiency before, all had been reported as high-yielding constructs [8,10,12]. To establish whether the same comparative patterns of yield and transduction efficiency were apparent at later timepoints in the production protocol, a time course including all three variants was performed, with samples harvested at 72, 96 and 120 h. Repeat analysis by qPCR/ELISA and transduction/flow cytometry confirmed this to be the case (Supplementary Materials Figures S2–S4).

### 2.2. VP Ratio Analysis

**Figure 3:**
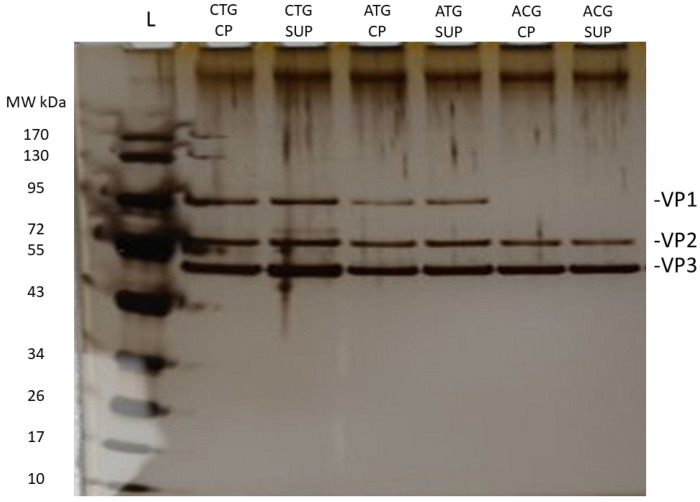
Equal protein amounts of all rAAV5 vector samples obtained post-harvest and affinity purification, including cell pellet and supernatant, were semi-quantitatively analyzed by silver-stained sodium dodecyl sulphate–polyacrylamide gel electrophoresis (SDS-PAGE). Details of the experimental conditions are provided in Section 3. Gels were used solely to visualize changes in VP protein abundances across different samples.

**Figure 4:**
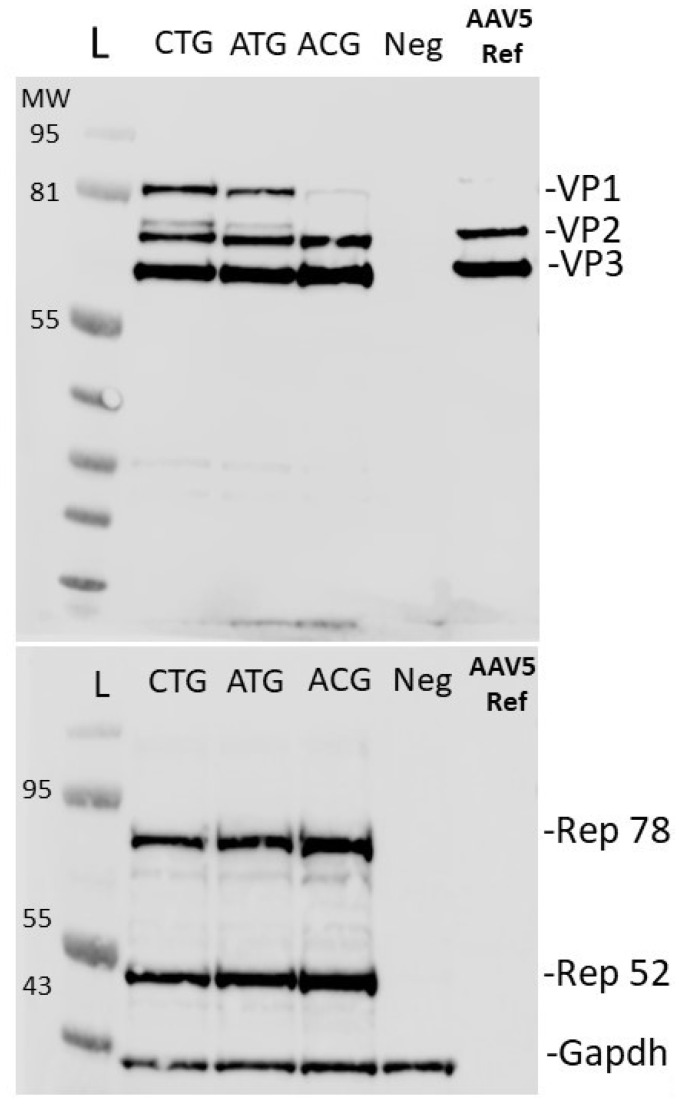
Anti-VP and anti-Rep Western blot. Protein samples were prepared from 1 mL of AAV-producing Sf9 cells using either CTG, ATG or ACG *cap5* gene start codons (at 72 h post-transduction). (**Above**) Samples probed with anti-AAV5 VP1/2/3. (**Below**) samples probed with anti-AAV2 Replicase. AAV5 Ref sample constitutes a HEK-293 produced and purified virus donated by Pharmaron-UK. Replicase should not be present in purified virus samples as it is not incorporated into the viral capsid. Gels were used solely to visualize changes in VP protein abundances across different samples.

Capsid protein stoichiometry and, in particular, increased abundance of VP1 have been implicated in reduced packaging efficiency [8,10,12,14,15]. Therefore, to analyze how our potency and production data correlated with the VP ratios, samples were initially run on a denaturing SDS-PAGE and silver-stained to assess the levels of each VP semi-quantitatively (Figure 3). Western blot analysis of lysates from BV-transduced Sf9 cells revealed a similar pattern of Rep expression (Figure 4) when comparing the three variants; however, the ACG sample resulted in visibly lower expression of VP1. This could also explain the lack of transduction efficiency due to insufficient VP1-linked phospholipase activity to escape the endosome, demonstrated by the ACG sample [15,17].

**Figure 5:**
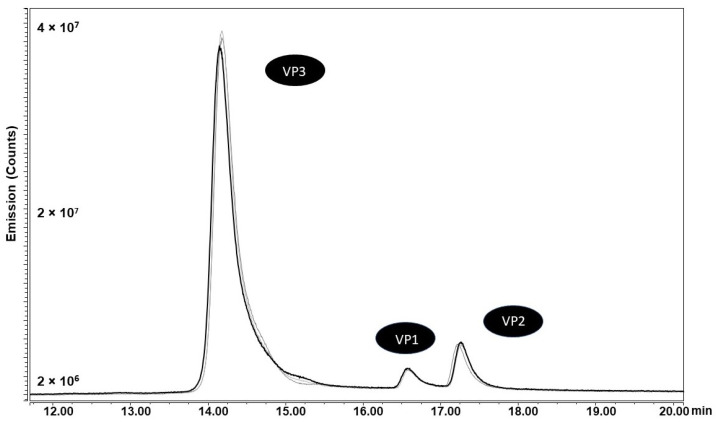
VP separation profiles (*n* = 3) obtained for the ACG CP rAAV5 variant during HILIC-FLR using DFA as an ion pairing agent; similar VP retention times were obtained for all samples.

Changing the *cap5* gene start codon to ACG resulted in visibly reduced VP1 expression compared to CTG and ATG start codon samples. Since SDS-PAGE and Western blotting are only semi-quantitative methods to estimate VP levels, we set out to develop a quantitative LC workflow to accurately determine VP ratios. Using a hydrophilic interaction liquid chromatography with fluorescent detection (HILIC-FLR)-based separation approach as first described by Liu et al. in 2020 [18], we developed an AAV5-specific gradient for the separation of VP proteins using difluoroacetic acid (DFA) as a mobile phase modifier, slightly adapted from the method described by Smith et al. [19]. After separation (Figure 5), ratios were calculated based on FLR peak areas and an assumption that the sum of VP1, 2 and 3 peaks accounted for 60 protein copies in the capsid, whereby a theoretical ratio sum of 12 (1:1:10, VP1/VP2/VP3) was used for the quantitative estimation of VP ratios [19] (full data available in Supplementary Materials Table S1).

**Figure 6:**
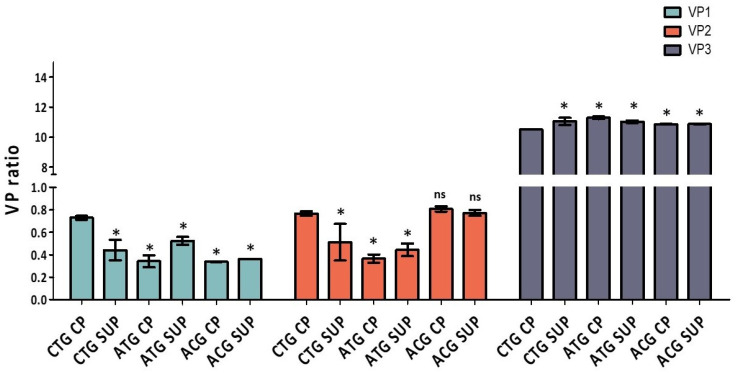
VP protein abundance levels per rAAV based on a total theoretical value of 12 (1:1:10 = 12, VP1:2:3) calculated using peak areas derived from FLR detection during HILIC separation. One way ANOVA was run for each VP protein using GraphPad Prism 5, followed by a post hoc Tukey’s multiple comparison test. Statistically significant values are identified as (*) *p* < 0.05 and (ns) is used for non-statistically significant results in comparison with CTG CP. For all individual comparisons, refer to statistical analysis results in the Supplementary Materials.

The ACG start codon was found to have the lowest amount of VP1 and also the lowest ratios of VP1/VP2 and VP1/VP3, which was in agreement with the profiles seen in the SDS-PAGE analysis for this construct (Table 1). The other variants had similar ratios between VP1 and VP2, although a higher abundance of VP1 and VP2 proteins was noted for the CTG variant in comparison to ATG (Figure 6 and Table 1).

Evidence that a low abundance of VP1 in AAV particles has the potential to negatively affect the potency of the vector has been extensively reported [13,14,15,20,21]. In addition, it has been suggested that the stoichiometry of the three VPs is crucial for vector transduction efficiency [8]. This observation correlates positively with our transduction efficiency data, which showed that the least infective variant was the ACG-containing vector, which was also the construct with the lowest VP1/VP2 ratio (Figure 2 and Figure 6 and Table 1).

Moreover, Bosma et al. reported that high levels of VP3 were associated with poor transduction, both in vitro and in vivo, highlighting a negative correlation between VP3 levels and potency. Excessive incorporation of VP3 decreased potency, even when the VP1/VP2 ratio was in balance. They found that the most potent construct had a 1:1 VP1/VP2 ratio and significantly lower levels of VP3, suggesting that an increased VP1/VP3 ratio would generate an AAV preparation where less administered particles are required to achieve the same levels of transduction efficiency, i.e., a lower dose. This observation was directly linked with an increase in the presence of the phospholipase 2 domain unique to the VP1 sequence, which has been shown to be responsible for the escape of the AAV virus from the endosome [17,22].

By this logic, CTG-derived vectors should display higher transduction efficiency levels than ATG, especially CTG CP. However, even though transduction efficiency levels reported for both rAAVs were similar (Figure 2), our results indicate that ATG is slightly more transduction-competent when compared to CTG CP for both CP and SUP. Thus, we set out to investigate whether it is possible that other factors, such as genome packaging efficiency, also affect viral transduction efficiency.

### 2.3. Packaging Efficiency

A multimethod comparison approach to explore the capabilities of novel LC and MS methodologies for determining full/empty ratios in rAAV was adopted. Ultracentrifugation is widely used for empty and full capsid separation, but it relies on large sample amounts and is a very time-consuming method [9].

The methods applied here have a clear advantage as they are very sensitive and results can be obtained in a much shorter timeframe [23]. To determine the extent of successful genome packaging in the samples investigated, we set out to compare two different methods using either LC or MS. As previously described and validated in Strasser et al. [23], anion exchange-liquid chromatography (AEX-LC) and MS are both well-suited to determine empty/full (E/F) capsid ratios, with results showing good correlation. In this study, we developed a pH gradient-based AEX-LC separation using low ionic strength buffers with a pH in the range of 10.3 to 2.6. These results were then compared with data obtained using a Native MS approach as described by Strasser et al. [23].

#### 2.3.1. AEX-LC

**Figure 7:**
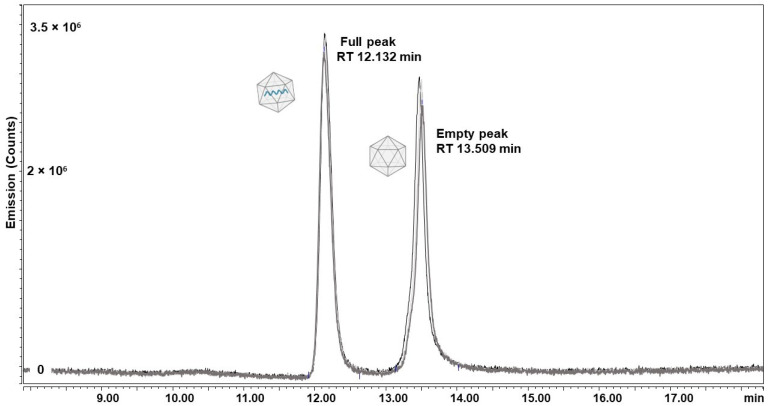
Chromatogram obtained by AEX with FLR detection of the ACG CP variant of rAAV5 (*n* = 3). Full capsid samples are indicated by the capsid schematic containing a DNA strand, whereas an empty schematic represents empty capsids. Peak retention times are also displayed. To ensure better visualization, only retention time regions of interest are presented.

**Figure 8:**
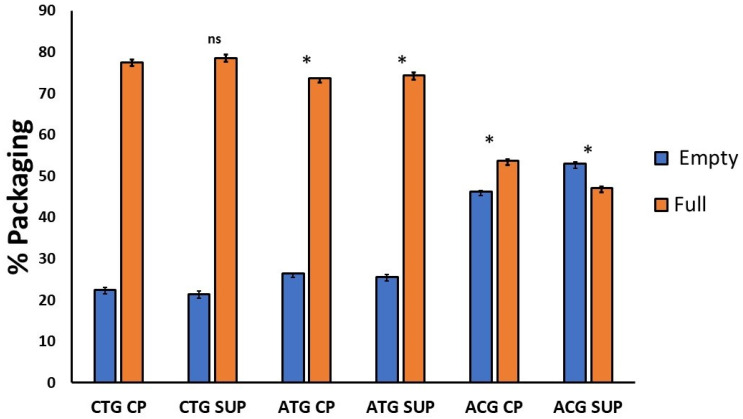
Percentage of empty and full capsids quantified by AEX-LC. Individual empty and full peak areas detected by FLR were integrated and used for the percentage calculation. One way ANOVA was run for each dataset using GraphPad Prism 5, followed by a post hoc Tukey’s multiple comparison test. Statistically significant values are identified as (*) *p* < 0.05 and (ns) is used for non-statistically significant results in comparison with CTG CP. For all individual comparisons, refer to statistical analysis results in the Supplementary Materials.

AEX-LC separation was performed on all six samples. An exemplar FLR trace showing the baseline separation of empty and full particles of ACG CP achieved by pH-gradient separation is displayed in Figure 7. With close to 80%, the greatest portion of full capsids was found in the CTG-containing samples, in both cell pellet- and supernatant-derived material. The percentage of full capsids present in constructs containing the ATG start codon was also above 70%; the ACG variant featured the lowest packaging efficiency, with ~50–55% of capsids containing a genome (Figure 8, full table of results is available in Supplementary Materials Table S2).

#### 2.3.2. Native MS

**Figure 9:**
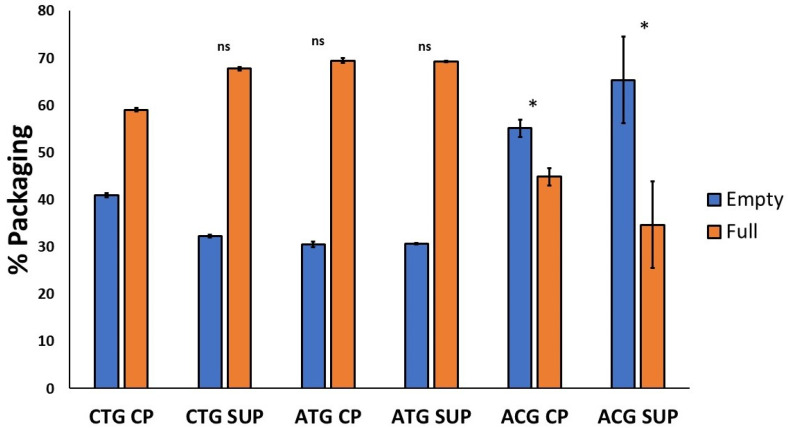
Percentage of empty and full capsids quantified by native MS. Signals in different *m*/*z* region corresponding to empty and full capsids were used for the percentage calculation. One way ANOVA was run for each dataset using GraphPad Prism 5, followed by a post hoc Tukey’s multiple comparison test. Statistically significant values are identified as (*) *p* < 0.05 and (ns) is used for non-statistically significant results in comparison with CTG CP. For all individual comparisons, refer to statistical analysis results in the Supplementary Materials.

Previously, it was demonstrated that full and empty AAV particles show the same charge state distribution when subjected to direct infusion-governed mass spectrometry under native conditions. The large mass discrepancy between full and empty species thereby facilitates signals in a different *m*/*z* region that allows for their use as a quantitative measure [23]. The same was observed in the current dataset, with full and empty particles populating *m*/*z* regions of around 21,000 and 30,000, respectively. Figure 9 shows the results of the quantitative packaging analysis using native MS. The highest proportion of full capsids was obtained using the ATG start codon, which was closely followed by CTG. The ACG construct was confirmed as the variant yielding the lowest percentage of full capsids. Full/empty quantitation by AEX featured the same trend, albeit the general proportion of empty capsids appeared lower when compared to the results from native MS analysis.

#### 2.3.3. Packaging Assessment by Orbitrap-Based Charge Detection Mass Spectrometry (CDMS)

While native MS analysis allows for the differentiation of full and empty particles and their quantitation, CDMS enables the direct mass assignment of capsid species and the determination of their cargo size (Table 2). In doing so, not only the masses of full and empty particles become accessible but potentially also other forms may be resolved if present, such as over- or underpackaged capsids [24,25,26,27,28]. Measurements were performed for all samples and ATG showed the lowest mass deviation, indicating correct packaging.

Understanding the packaging efficiency of vectors is critical for determining clinical dose and assuring the safety of the drug product since empty or incorrectly packed vectors are considered process impurities. Furthermore, the encapsidation process of full or partial AAV genomes may lead to capsid conformational changes, especially those involving the capsid VP1 *N*-terminus, which contains a phospholipase A2 domain, and two nuclear localization signals that can be reversibly externalized through pH induction [29]. Such conformational changes in capsid structure expose both the phospholipase A2 domain and nuclear localization signal domains and facilitate endosome escape and viral infection [30]. Therefore, not only do VP stoichiometry and high relative VP1 abundance play a role in transduction efficiency but also the packaging state of the viral particle.

Furthermore, it has been noted in AAV8 preparations that an excessive increase in VP1 in the capsid stoichiometry results in reduced packaging efficiency, meaning that too much VP1 can also increase the percentage of empty capsids [31]. Our AEX-LC data corroborate such observations, showing a higher percentage of empty material present for the ATG start codon when compared with CTG. ATG also contains a very similar (CP) or higher (SUP) ratio of VP1 in relation to VP2 than CTG (Table 1). However, slight method-dependent variation was observed between the AEX pH gradient data and the MS data when determining whether ATG or CTG contained the highest number of empty capsids. Even though the MS data did not identify statistically significant differences between CTG and ATG, further studies are necessary to understand whether the high temperatures used during MS acquisition or the low pH used during pH-based empty and full chromatographic separation could be impacting AAV5 capsid integrity and account for the variations observed between the two methods.

Additionally, the direct correlation between the excessive incorporation of VP1 and the high abundance of empty capsids reported previously [31] could not be verified in our study. None of the constructs produced a VP ratio close to theoretical values of 1:1:10 or showed higher levels of VP1 over the other capsid proteins. Nonetheless, the lowest level of VP1 incorporation and the highest levels of empty material observed in ACG contradict that report and align with Bosma et al. in concluding that no direct correlation between altered levels of VP1 and total empty/full ratio was found during rAAV production.

Our productivity results demonstrate that the highest yields were obtained with ACG, a construct that, as discussed, had the lowest incorporation of VP1 and percentage of full capsids. Notably, in this case, quantity did not correlate with the quality of capsids. On the other hand, CTG resulted in the lowest yield among the three rAAVs. It has been postulated that the leaky ribosomal scanning mechanism generating these capsids in insect cells can lower or delay VP1 production when using a CTG start codon and, ultimately, have a negative effect on the overall yield [8,31]. This could explain the low yield obtained for CTG in our experiments and expands on what was first described for AAV8 and now also for AAV5. Further studies would be required to elucidate the potential mechanisms guiding intracellular trafficking and VP and DNA incorporation [32] during rAAV production. However, the scope of this study was not to interrogate the biological mechanisms underpinning AAV production but to demonstrate the applicability of a complete characterization workflow for the critical quality attribute (CQA) analysis of rAAVs using novel LC and MS techniques. The workflows demonstrated here have the potential to inform and improve the process development and manufacturing of AAV-based gene therapy products.

## 3. Materials and Methods

### 3.1. Plasmid and BV Construction

The pFastBac_dual plasmid was acquired from Gibco as part of the Bac-to-Bac system. An AAV2-ITR-flanked *GFP* expression cassette was amplified from pAAV-gfp (addgene #32395) and ligated into pFastBac_dual using KpnI and MfeI restriction sites. pFastBac_cap5_rep2, which contained one of each of the three AAV5-derived cap5 gene variants (Figure 1) under control of the P10 promoter and the AAV2-derived rep2 gene under control of the polyhedrin promoter, was produced by Genscript. PacI and NheI sites were used to insert cap5 genes, with start codons modified by PCR. Plasmid maps are shown in Supplementary Material (Figure S5). *E. coli* DH 10Bac cells containing the bacmid were transformed with each plasmid to generate 2 recombinant bacmids according to the manufacturer’s instructions (Bac-to-Bac^®^ Baculovirus Expression System—Gibco (Grand Island, NY, USA) Cat. No. 10359-016). Sf9 cells were, in turn, transfected with these bacmids and the resulting recombinant baculoviruses (BVs) were collected and assayed for viral titre. BVs were stored at −80 °C.

### 3.2. Sf9 Cell Culture and Transductions

*Spodoptera frugiperda* Sf9 cells (Thermo Fisher Scientific (Waltham, MA, USA) Cat. 12659017) were maintained in suspension in Gibco Sf-900 III SFM (Cat. 12658027; Thermo Fisher Scientific), a chemically defined, serum-free insect cell culture medium. The cells were cultured between passages 3 and 20 in 125 mL Erlenmeyer flasks at 28 °C and agitated at 170 rpm in a New Brunswick™ S41i Incubator Shaker (Eppendorf, Hamburg, Germany).

#### Sf9 Transduction

Sf9 cells were seeded at 1 × 10^6^ cells per mL in 250 mL Erlenmeyer flasks, followed by transduction with both the gene of interest-containing baculovirus and the relevant Rep2/Cap5 baculovirus at a multiplicity of infection (MOI) of 0.3, which was found to generate sufficient AAV titers after testing MOIs of 15, 5, 3, 1 and 0.3.

### 3.3. AAV Harvest

After 72 h of incubation, cell viability and size were determined with a Luna cell counter. Next, 2 mM (final concentration) MgCl_2_ and 100 U/mL benzonase were added to the cells. Incubation continued for a further hour, followed by transfer to 50 mL Falcon tubes and centrifugation (16,000× *g*, 20 min, 4 °C). The supernatants were removed, passed through a 0.22 μm filter and stored at −80 °C prior to further purification. The cell pellets were resuspended in 10 mL Sf-900 III SFM media and then freeze/thawed three times by placing them alternately into liquid nitrogen and a water bath at 37 °C. After each thaw, the suspension was vortexed vigorously for 30 s. Then, 2 mM MgCl_2_ and 100 U/mL benzonase were added and the samples were incubated for a further 1 h at 37 °C in an 8% CO_2_ environment. Finally, the samples were centrifuged (16,000× *g*, 20 min, 4 °C). The supernatant was passed through a 0.22 μm filter and stored at −80 °C for further purification.

### 3.4. Viral Vector Purification

Harvested supernatants and cell pellets were stored at −80 °C and purified using an ÄKTA avant 150 FPLC system with a 5 mL POROS™ GoPure™ AAVX pre-packed column (0.8 × 10 cm) from Thermo Fisher Scientific (Paisley, UK). Samples were loaded at a flow rate of 1 mL/min, followed by a high salt wash step with 50 mM Tris, 1 M NaCl, 2 mM MgCl_2_ and 0.01% Pluronic at pH 7.5 for 10 column volumes (CVs) and a low pH wash step with 50 mM Tris, 0.2 M NaCl, 2 mM MgCl_2_ and 0.01% Pluronic at pH 4.0 for 10 CVs. The pH was equilibrated during 10 CVs using 50 mM Tris, 0.2 M NaCl, 2 mM MgCl_2_ and 0.01% Pluronic at pH 7.5 prior to the elution of the AAVs with 80% 100 mM glycine, 2 mM MgCl_2_ and 0.01% Pluronic at pH 2.0 for 10 CVs. The eluate was neutralized with 1.0 M Tris and then buffer-exchanged and concentrated in 0.01% Pluronic in 1× PBS solution using 100 kDa Amicon^®^ Ultra-0.5 filter devices (Merck, Darmstadt, Germany).

### 3.5. Western Blot and Silver Stain

Western blots were performed using Bolt 4 to 12%, Bis-Tris and 1.0 mm, mini-protein, 12-well gels run at 200 V for 34 min in MOPS 1× NuPAGE running buffer. A total of 20 μL of the sample was added to 10 μL of NuPAGE LDS sample buffer (4×), 4 μL of NuPAGE reducing agent (10×) and 6 μL of deionized water. Samples were heated to 85 °C for 10 min and then incubated at room temperature for 10 min before loading 30 μL of the sample. The Power Blotter-Semi-dry transfer system was used at 25 V for 10 min to transfer samples onto a nitrocellulose membrane. Blocking was performed using Odyssey Blocking buffer (TBS) for 1 h. The primary antibodies (anti-AAV2 Replicase mouse monoclonal, 303.9, 61069 Progen and anti-AAV5 VP1/VP2/VP3 mouse monoclonal, B1, 61058 Progen) were diluted 1:250 and incubated with the membrane overnight in Odyssey Blocking Buffer (TBS), 0.1% tween-20. Washes were performed 4 times (5 min each) using PBS with 0.1% tween-20 buffer. The secondary antibody (Goat anti-Mouse IgG Secondary Antibody IRDye 680 CW) was diluted 1:10,000 and applied for 1 h In Odyssey Blocking Buffer (TBS), 0.1% tween-20, followed by a further wash step. Imaging was performed using a LICOR system.

Silver staining was performed using the Pierce silver stain kit (Thermo Fisher Scientific Cat. 24612) by washing the gel twice for 5 min in ultrapure water, followed by 2 fixation steps in a 30% *v*/*v* ethanol, 10% *v*/*v* acetic acid solution for 15 min. The gel was washed twice for 5 min in 10% *v*/*v* ethanol and then twice for 5 min in ultrapure water. A total of 50 μL of sensitizer solution was added to 25 mL water and then used to sensitize the gel for 1 min, followed by 2 washes in ultrapure water for 1 min each. The stain working solution (0.5 mL Enhancer with 25 mL stain) was prepared and added to the gel for 30 min. The gel was washed twice for 20 s in ultrapure water before adding Developer working solution (0.5 mL Enhancer and 25 mL Developer). The gel was developed for 2–3 min until bands appeared and then stopped by adding 5% *v*/*v* acetic acid for 10 min.

### 3.6. Titre Determination by qPCR and Capsid ELISA

#### 3.6.1. qPCR

A total of 10 µL of AAV samples were pre-treated to remove DNA not contained in viral capsids by incubation in 80 µL nuclease-free water with 2 mM MgCl_2_ and 1.25 U/µL of benzonase at 37 °C for 30 min. A total of 10 µL of 0.05 M EDTA was then added, and incubation continued at 68 °C for 10 min, followed by a 4 °C hold. A solution of 6 U/µL Proteinase K in nuclease-free water was prepared. A total of 10 µL of the benzonase-treated AAV sample was added to 90 µL of Proteinase K solution and vortexed to obtain a 1:100 dilution. A total of 10 µL of the 1:100 dilution was then added to a further 90 µL of Proteinase K solution and vortexed to obtain a 1:1000 dilution. The 1:100 and 1:1000 dilutions were incubated at 50 °C for 1 h, followed by 95 °C for 20 min and a 4 °C hold. qPCR was performed on an Applied Biosystems QuantStudio 3 instrument, with primers targeting the AAV ITR region using the absolute quantitation method (primer sequences subject to company IP). A standard curve was prepared using a purified PCR product of the ITR region, which was measured on a spectrophotometer to determine concentration and calculate the copy number. This was then 10-fold serially diluted from 4 × 10^8^ to 4 × 10^3^ total copies. The efficiency ranged from 90–100%. A total of 4 µL of benzonase/proteinase-treated AAV sample (100- and 1000-fold dilutions) was mixed with 1 µL each of 10 mM primers, 4 µL of nuclease-free water and 10 µL of Fast SYBR Green mastermix (Thermo Fisher Scientific). qPCR was performed with a 2 min/50 °C hold and then a 95 °C/10 min hold, followed by 40 cycles of 95 °C/15 s and 60 °C/1 min, with an autodelta setting of −0.1 °C per cycle. The Ct value was set to 0.4.

#### 3.6.2. ELISA

ELISA measurements were performed using a Progen AAV5 kit, according to the manufacturer’s instructions. Results were read on a plate reader at 450 and 650 nm wavelengths. One way ANOVA and Tukey’s multiple comparison tests were carried out using GraphPad Prism version 5.0.

### 3.7. Transduction Efficiency Assay

CHO-K1 cells were suspension-cultured in CHO-SFM with an anti-clumping agent. For transduction experiments, 1 × 10^5^ cells were seeded into each well of a 24-well plate and grown in DMEM + 10% FBS for 24 h before adding 22,000 vg/cell, followed by incubation for a further 72 h. Following incubation, the supernatant was removed and cells were resuspended in 4% formaldehyde/phosphate-buffered saline (PBS) for 15 min. After centrifugation (300× *g* for 5 min), cells were washed in PBS. The wash step was repeated twice. Flow cytometry was performed using a BD Accuri instrument. One way ANOVA and Tukey’s multiple comparison tests were carried out using GraphPad Prism.

### 3.8. Hydrophilic Interaction Liquid Chromatography (HILIC) Intact Separation of VP Proteins

AAV capsid protein separation was performed to determine VP ratios for each construct on a Vanquish™ Horizon ultra-high pressure liquid chromatograph (UHPLC) (Thermo Fisher Scientific, Germering, Germany), slightly modified from Smith et al., 2023 [19]. In brief, separation was obtained on a Waters ACQUITY UPLC Glycoprotein BEH Amide column (300 Å, 1.7 µm, 2.1 × 150 mm) (Waters Corporation, Milford, MA, USA, p/n: 186007963) using a flow rate of 0.1 mL/min and column temperature of 45 °C. The gradient started at 85% acetonitrile (ACN) and 15% water for 3.5 min, 85% ACN to 65% ACN over the next 0.1 min, and a hold at 65% ACN for 4.4 min, followed by a 21 min gradient from 65% to 45% ACN. Column wash and equilibration were carried out at a high flow rate prior to subsequent sample analysis. Fluorescence (FLR) detection was used to quantify peak areas of each separated VP protein. The sum of all 3 peak areas was used to calculate individual protein abundance levels and ratios in triplicates, assuming a theoretical ratio of 1:1:10 = 12. One way ANOVA and Tukey’s multiple comparison tests were carried out using GraphPad Prism.

### 3.9. Anion Exchange Liquid Chromatography (AEX-LC) Analysis of Empty and Full Capsids

Prior to liquid chromatography separation of empty and full rAAVs, samples were buffer-exchanged into aqueous ammonium acetate (100 mM, pH 6.8) using 100 kDa Amicon^®^ Ultra-0.5 filter devices (Merck, Darmstadt, Germany). Separation was performed using a ProPac SAX-10, 1 × 50 mm column (Thermo Fisher Scientific, Sunnyvale, CA, USA) using FLR detection (Ex280/Em340) on a Vanquish Horizon UHPLC system (Thermo Scientific, Germering, Germany). Buffer A was 20 mM ammonium bicarbonate and 15 mM ammonium hydroxide, pH 10, and buffer B was 15 mM formic acid and 30 mM acetic acid, pH 2.3. At a flow rate of 0.15 mL/min and column temperature of 30 °C, empty and full capsids were separated using the following conditions: samples were loaded onto the column at 0.1% B followed by an increase to 20% B over 1 min. Separation was carried out using a two-step gradient of 20–60% B over 4 min followed by a hold at 60% B for 2.9 min and a second ramp from 60 to 80% B over 8 min. A wash step at 90% B was carried out for 1.5 min and column re-equilibration at 0.1% B for 17.4 min. FLR-detected peak areas were used for calculating the percentage of empty and full capsids in triplicates. One way ANOVA and Tukey’s multiple comparison tests were carried out using GraphPad Prism.

### 3.10. Native and Charge Detection Mass Spectrometry of Empty and Full Capsid Ratios

Both mass spectrometry techniques were carried out by means of static nano-electrospray ionization-based direct infusion on a Q Exactive Ultra High Mass Range (UHMR) mass spectrometer (Thermo Scientific, Bremen, Germany). Native mass spectrometry experiments were performed as previously described by Strasser et al. [23]. Peak areas were used for calculating the percentage of empty and full capsids. One way ANOVA and Tukey’s multiple comparison tests were carried out using GraphPad Prism. Charge detection mass spectrometry was based on the Direct Mass Technology™ (DMT) mode provided by Thermo Scientific. Acquisition parameters are shown in detail in Table S3 of the Supplementary Materials. Raw files were analyzed in STORIboard 1.0 (Proteinaceous, Evanston, IL, USA). For charge assignment, an experimentally determined calibration curve with up to 80 charges was applied, which was further extrapolated up to 240 charges) [33]. Data processing was based on a processing method that was previously optimized for the analysis of large macromolecular entities such as AAVs.

## 4. Conclusions

In this work, the TwoBac/Sf9 system was used to produce rAAV5 variants with either ATG, ACG or CTG as the start codon of the *cap* gene. A complete characterization workflow from purification to titer determination, VP ratio analysis and capsid packaging assessment using HPLC and MS, including HILIC, AEX and native MS, was implemented in this work. By providing a detailed characterization of the expressed viral capsid-based drug substance, our workflow has the potential to aid the improvement of AAV production-related processes. The ACG start codon, while being the most productive construct as determined using traditional analytics (AAV capsid ELISA), resulted in overall reduced transduction efficiency assessments, potentially due to lower levels of correct packaging, high quantities of empty capsids or lower levels of VP1. The relationship between VP stoichiometry and packaging efficiency requires further investigation, as does the intracellular processing of VPs. However, it was observed that a low VP1/VP2 ratio correlated with significantly decreased viral transduction efficiency, even when capsid yield was high, as evidenced by the ACG start codon variant. 

Among the constructs analyzed, the mutant containing the ATG start codon as first described by Kondratov et al. [12] seemed to be the most efficient, generating higher titers than CTG while still maintaining a VP stoichiometry close to theoretically ideal values, high transduction efficiency and a high portion of full capsids with the lowest mass deviation, indicating correct packaging.

It is anticipated that this characterization workflow will be beneficial in the process development and manufacturing of AAV, allowing for the rapid monitoring of critical quality attributes and progressing the development of more productive, safer and increasingly effective rAAVs.

## Figures and Tables

**Table 1:** VP abundance levels and protein/protein ratios calculated based on peak areas obtained during HILIC-FLR analysis. The theoretical ratio of 1:1:10 = 12 was used to calculate values for each protein based on total and individual peak areas.

	VP1	VP2	VP3	VP1/VP2	VP1/VP3	VP2/VP3
CTG CP	0.7	0.8	10.5	0.95	0.07	0.07
CTG SUP	0.4	0.5	11.0	0.86	0.04	0.05
ATG CP	0.3	0.4	11.3	0.94	0.03	0.03
ATG SUP	0.5	0.4	11.0	1.18	0.05	0.04
ACG CP	0.3	0.8	10.9	0.42	0.03	0.07
ACG SUP	0.4	0.8	10.9	0.47	0.03	0.07

**Table 2:** Mass of empty and full capsids calculated for each rAAV sample using Orbitrap-based CDMS.

Sample	Mass kDa Empty	Mass kDa Full	Δm E/F kDa	Mass Deviation % *
CTG CP	3742.44	4884.41	1141.97	12.3
CTG SUP	3713.26	4906.12	1192.85	17.3
ATG CP	3654.10	4755.79	1101.70	8.3
ATG SUP	3653.32	4714.71	1061.39	4.4
ACG CP	3583.57	4737.56	1153.98	13.5
ACG SUP	3594.25	4525.09	930.839	−8.5

* Mass deviation [%] = deviation from expected mass shift. A full-length single-stranded DNA insert should have a molecular weight of 1017 kDa.

## Data Availability

Data is contained within the article and Supplementary Material.

## Supplementary Materials

The following supporting information can be downloaded at https://www.mdpi.com/article/10.3390/ijms25052785/s1.
