# Supplementary material for: A Direct Comparison of rAAV5 Variants Derived from the Baculovirus Expression System Using LC-MS Workflows Demonstrates Key Differences in Overall Production Yield, Product Quality and Vector Efficiency
Source: Int J Mol Sci. 2024 Feb 28;25(5):2785. doi: 10.3390/ijms25052785 (PMC10932283; doi:10.3390/ijms25052785)
Supplement: Supplementary file 1 [file ijms-25-02785-s001.zip › Supplementary Material Tables and Figures.pdf]

Article

A Direct Comparison of rAAV5 Variants Derived from the Baculovirus Expression System Using LC-MS Workflows Demonstrates Key Differences in Overall Production Yield, Product Quality and Vector Efficiency

Felipe Guapo [1,†], Nicholas Donohue [1,†], Lisa Strasser [1], Stefano Boi [1], Florian Füssl [1], Alana Rainbow-Fletcher [2], Paul Getty [2], Ian Anderson [2], Niall Barron [1,3,*] and Jonathan Bones [1,3,*]

[1] National Institute for Bioprocessing Research and Training, Foster Avenue, Mount Merrion, Blackrock, A94 X099 Dublin, Ireland
[2] Pharmaron, 12 Estuary Banks, Speke, Liverpool L24 8RB, UK
[3] School of Chemical and Bioprocess Engineering, University College Dublin, Belfield, Dublin 4, D04 V1W8 Dublin, Ireland
[*] Correspondence: niall.barron@nibrt.ie (N.B.); jonathan.bones@nibrt.ie (J.B.)
[†] These authors contributed equally to this work.

Supplementary Material

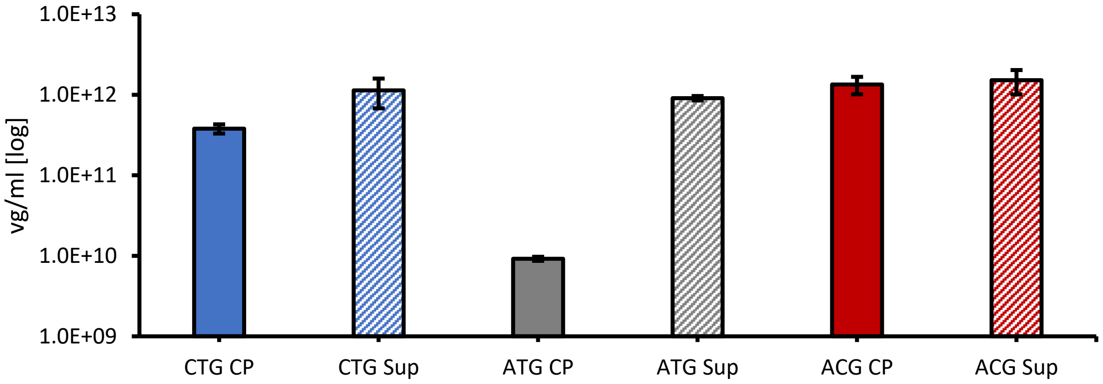

Figure S1: Total yield measured by qPCR representing material generated from recombinant and wild type AAV5 with harvest taking place after 72hr.

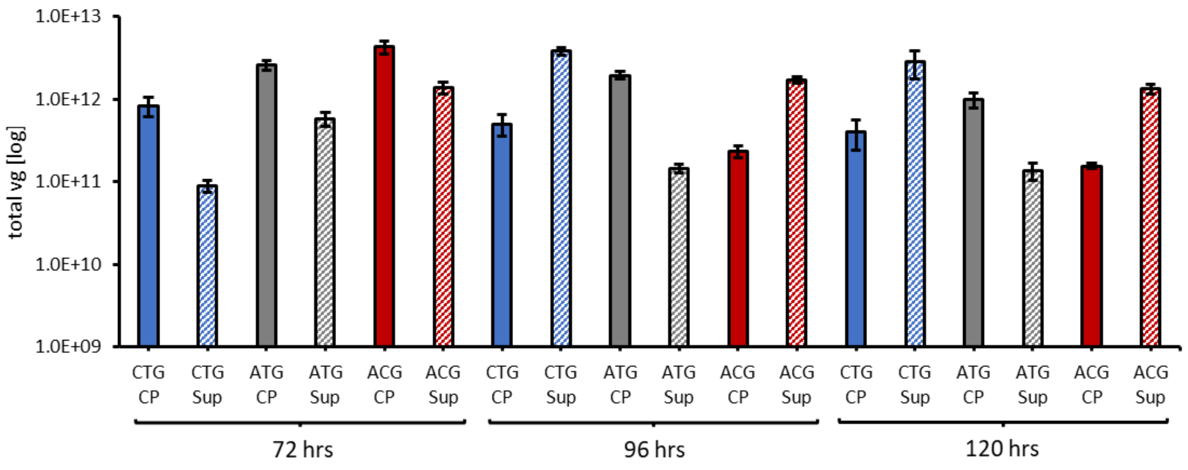

Figure S2: Total yield measured by qPCR representing material generated from recombinant and wild type AAV5 repeats, with harvest taking place after 72, 96 and 120 hr.

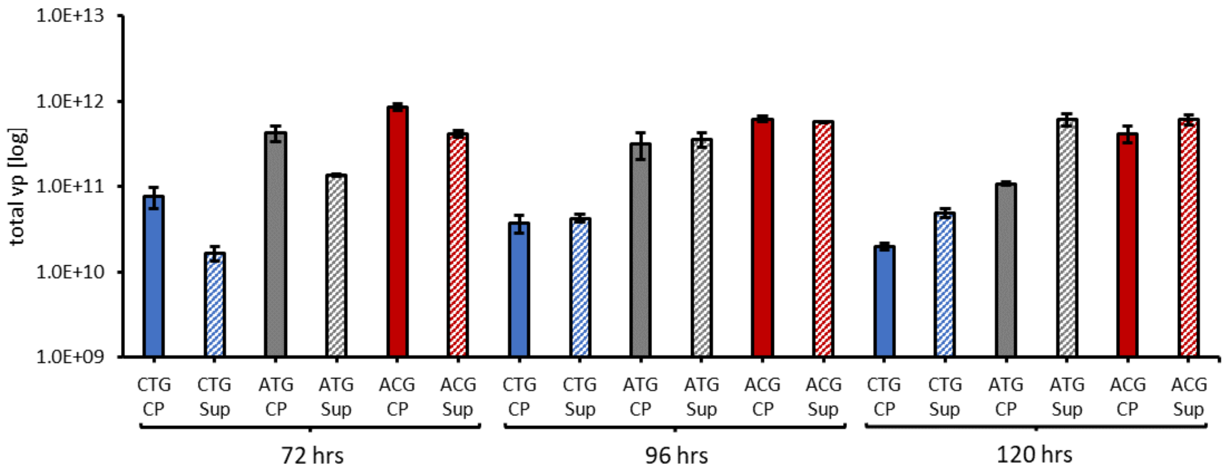

Figure S3: Total titre/yield measured by capsid ELISA representing material generated from recombinant and wild type AAV5, with harvest taking place after 72, 96 and 120 hr.

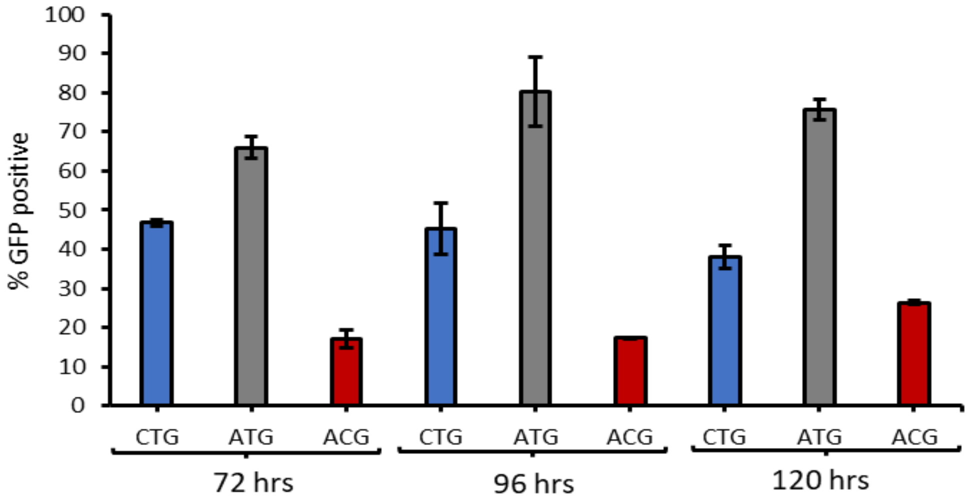

Figure S4: *In vitro* potency assay of wild type and variant capsids in CHO-K1 cells. Measurements taken using the cell pellet samples only.

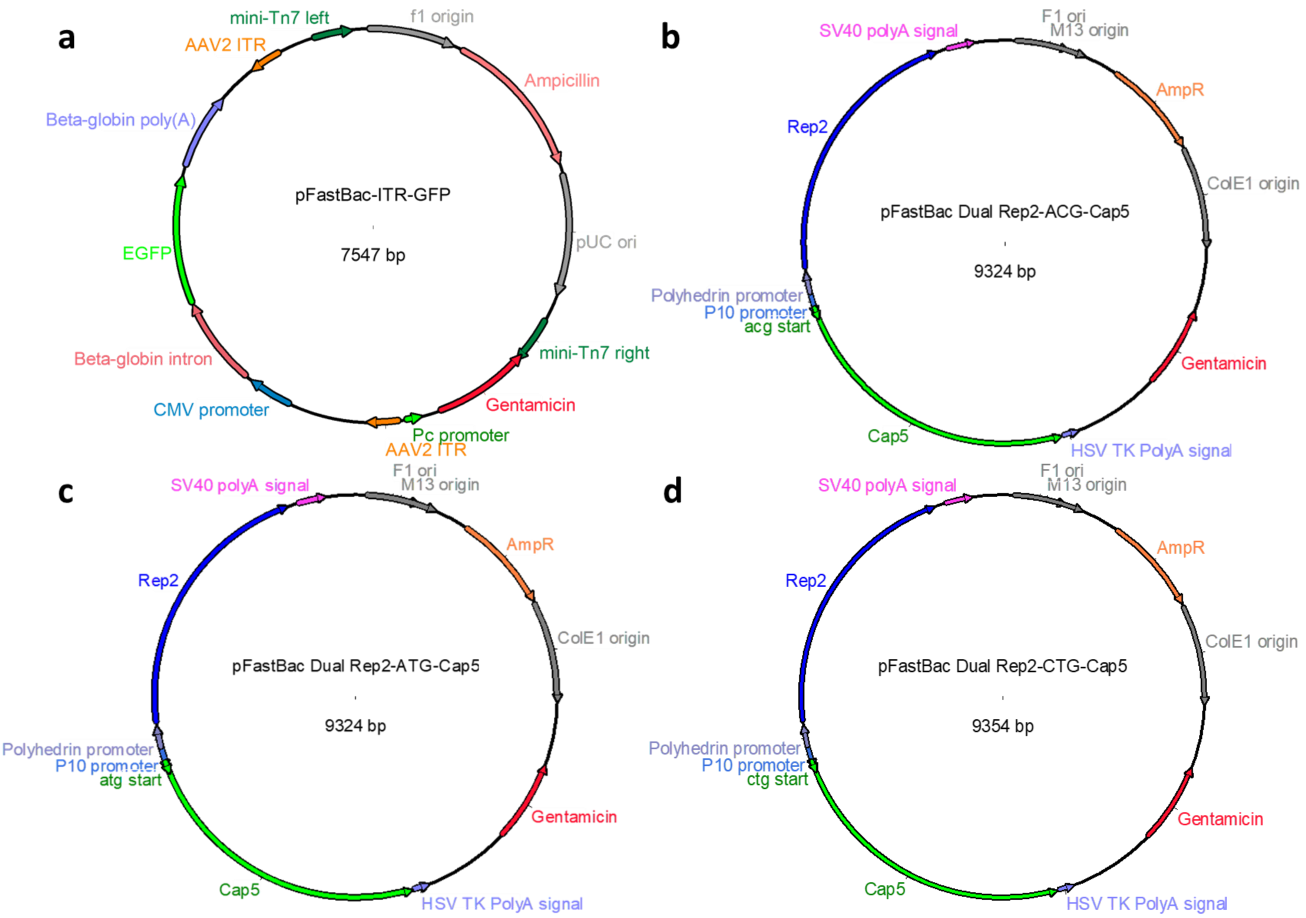

Figure S5: Plasmid maps of constructs used to generate Baculovirus, which were subsequently used to produce AAV. **a,** gene-of-interest carrying construct containing the GFP gene flanked by AAV2 ITR sequences. **b,** ACG start codon variant of the Rep/Cap carrying construct. **c,** ATG start codon variant of the Rep/Cap carrying construct. **d,** CTG start codon variant of the Rep/Cap carrying construct. GenBank accession numbers are as follows: BankIt2794929pFastBac-ITR-GFP: PP328554, BankIt2794929pFastBacDual-Rep2-ACG-Cap5: PP328555 BankIt2794929pFastBacDual-Rep2-ATG-Cap5: PP328556, BankIt2794929pFastBacDual-Rep2-CTG-Cap5: PP328557.

Table S1: VP ratio analysis per replicate of rAAV based on a total ratio theoretical value of 12 (1:1:10, VP1:2:3) calculated using peak area quantitation based on the FLR signal during HILIC separation and used to plot bar charts available in the main text.

	**VP1**					**VP2**					**VP3**				
	Rep 1	*Rep 2*	*Rep 3*	*Average*	*SD*	*Rep 1*	*Rep 2*	*Rep 3*	*Average*	*SD*	*Rep 1*	*Rep 2*	*Rep 3*	*Average*	*SD*
**CTG CP**	0.710	0.742	0.736	0.729	0.014	0.786	0.749	0.767	0.767	0.015	10.504	10.509	10.497	10.504	0.005
**CTG SUP**	0.370	0.413	0.543	0.442	0.074	0.486	0.364	0.686	0.512	0.133	11.144	11.223	10.771	11.046	0.197
**ATG CP**	0.396	0.339	0.291	0.342	0.043	0.403	0.365	0.328	0.366	0.030	11.201	11.295	11.380	11.292	0.073
**ATG SUP**	0.547	0.484	0.540	0.524	0.028	0.445	0.388	0.500	0.444	0.046	11.008	11.128	10.960	11.032	0.071
**ACG CP**	0.338	0.338	0.336	0.337	0.001	0.785	0.805	0.832	0.808	0.019	10.877	10.857	10.831	10.855	0.018
**ACG SUP**	0.365	0.362	0.361	0.363	0.002	0.790	0.784	0.744	0.772	0.020	10.845	10.855	10.895	10.865	0.022

Table S2: Percentage of empty and full capsids per replicate of rAAV quantified by native mass spectrometry and AEX-LC and used to plot bar charts available in the main text.

	Native MS									
	Rep 1	*Rep 2*	*Rep 3*	*Rep 1*	*Rep 2*	*Rep 3*	*Average*		*SD*	
		%E			*%F*		*%E*	*%F*	*%E*	*%F*
**CTG CP**	41.30	41.20	40.40	58.70	58.80	59.60	40.97	59.03	0.40	0.40
**CTG SUP**	31.90	32.70	32.20	68.10	67.30	67.80	32.27	67.73	0.33	0.33
**ATG CP**	30.30	31.30	30.00	69.70	68.70	70.00	30.53	69.47	0.56	0.56
**ATG SUP**	30.90	30.60	30.60	69.10	69.40	69.40	30.70	69.30	0.14	0.14
**ACG CP**	52.60	56.00	56.80	47.40	44.00	43.20	55.13	44.87	1.82	1.82
**ACG SUP**	52.40	71.70	71.90	47.60	28.30	28.10	65.33	34.67	9.15	9.15
	AEX-LC-FLR									
**CTG CP**	23.25	22.16	21.92	76.74	77.84	78.08	22.44	77.55	0.58	0.58
**CTG SUP**	21.12	22.53	20.65	78.88	77.47	79.35	21.43	78.57	0.80	0.80
**ATG CP**	26.34	26.31	26.63	73.66	73.69	73.37	26.43	73.57	0.14	0.14
**ATG SUP**	26.13	24.76	26	73.87	75.24	74	25.63	74.37	0.62	0.62
**ACG CP**	46.22	46.66	46.01	53.78	53.34	53.99	46.30	53.70	0.27	0.27
**ACG SUP**	52.8	53.53	52.52	47.2	46.47	47.48	52.95	47.05	0.43	0.43

Table S3: MS acquisition parameters used for charge detection mass spectrometry based on the Direct Mass Technology™ (DMT) mode provided by Thermo Scientific on a Q Exactive Ultra High Mass Range (UHMR) mass spectrometer (Thermo Scientific, Bremen, Germany).

**Parameter**	**Setting**
Spray voltage	0.9 kV
Capillary temperature	275°C
S-lens RF level	200
Resolution setting at m/z 400	50,000
Trapping gas	Nitrogen
UHV pressure (mbar)	4.6e-10 mab
IST desolvation voltage	-150 V
Averaging	Off
Detector m/z optimization	High
Ion transfer target	High
Ion Injection	IT = 200
